# School-based interventions for resilience in children and adolescents: a systematic review and meta-analysis of randomized controlled trials

**DOI:** 10.3389/fpsyt.2025.1594658

**Published:** 2025-05-19

**Authors:** Chenyi Cai, Zhengyang Mei, Zirui Wang, Shi Luo

**Affiliations:** ^1^ School of Physical Education, Southwest University, Chongqing, China; ^2^ College of Horticulture and Landscape Architecture, Southwest University, Chongqing, China

**Keywords:** school-based interventions, resilience, children, adolescents, mental health

## Abstract

**Objective:**

This systematic review and meta-analysis aimed to evaluate the overall efficacy of school-based interventions (SBIs) in promoting resilience in children and adolescents and to provide evidence for advancing mental health care for children and adolescents.

**Methods:**

A search was conducted in seven electronic databases, including PubMed, Embase, EBSCOhost, Scopus, Web of Science, APA PsycINFO, and Google Scholar. The Revised Cochrane risk-of-bias tool for randomized trials (RoB 2) was used for the quality appraisal. The standardized mean difference (SMD; Cohen’s d) combined with 95% confidence intervals (CIs) was used to pool the effect sizes.

**Results:**

A total of 38 RCTs involving 15,730 participants were included in the systematic review, 21 of which were selected for inclusion in the meta-analysis. In terms of quality appraisal, the included trials were classified as having low risk, some concerns, or high risk, with proportions of 5.2%, 71.1%, and 23.7%, respectively. The pairwise meta-analyses indicated that SBIs significantly enhanced resilience in children and adolescents compared to the control group (SMD = 0.17, 95% Cl 0.06–0.29, *p* < 0.01).

**Conclusions:**

SBIs have a positive effect on the resilience of children and adolescents. In the context of limited medical resources, SBIs could serve as a promising approach to promote the ability of children and adolescents to adapt to stressors. Given the considerable heterogeneity identified, SBIs should be personalized on the basis of variations in demographic characteristics, intervention implementation, and actual dose-response to improve the overall well-being of children and adolescents and reduce the risk of maladaptive psychological and behavioral responses.

## 1 Introduction

Resilience refers to an individual’s ability to cope and recover effectively in the face of setbacks and adversity and maintain normal physiological function and psychological health (1, 2). Resilience is a dynamic process for adapting to stressful situations, characterized by continuous development over time (3–5). The level of resilience is a crucial indicator for assessing an individual’s physical and mental development in response to stressors (6). Children and adolescents are in a critical stage of physical and mental development, making them susceptible to stressors from family, school, and peers, including parental divorce (7), academic pressure (8), teacher-student relationships (9), and school bullying (10, 11). Resilience can effectively help them overcome stressful situations, maintain psychological balance, and promote their positive development. According to the American Medical Association, approximately 293 million children and adolescents worldwide suffer from at least one mental disorder, such as anxiety, depression, and schizophrenia (12). To address this challenge, developing resilience is considered a promising approach (2, 13, 14). Evidence suggests that children and adolescents with higher resilience levels tend to respond positively to stressful situations by adopting adaptive coping strategies. This helps them reduce the risk of developing negative emotions such as anxiety and depression and promotes their overall mental health and welfare (15–17). By contrast, children and adolescents with lower resilience levels are more likely to exhibit problem behaviors, including smartphone addiction (18), violent tendencies (19), attention deficit hyperactivity disorder (20), illicit drug use, and smoking and alcohol abuse (21). The findings suggest that resilience, as an important psychological resource, can effectively reduce the negative impact of stressors on the growth process of children and adolescents, thereby promoting their physical and mental health development and enhancing their ability to adapt to and cope with stressful situations.

Interventions to promote resilience have been developed in a variety of settings to reduce mental health problems in children and adolescents, including families (22), communities (23), and hospitals (24). Family-based interventions aimed to improve family cohesion by strengthening positive communication between students and parents, thereby enhancing students’ resilience and reducing the occurrence of mental health disorders and issues during adolescence (22). Community-based interventions aimed to improve family economic conditions and provide children and adolescents with a stronger social support network, thereby promoting the development of their resilience (23). Hospital-based interventions aimed at helping patients build resilience through the care provided by healthcare professionals, enabling them to cope more effectively with stressors (25). However, interventions implemented in these environments may struggle to reach every child and adolescent. In this context, one of the most powerful tools for addressing the inequality in access to interventions is the implementation of school-based interventions (SBIs) to promote the physical and mental health of children and adolescents. SBIs are defined as any program, intervention, or strategy applied within the school environment aimed at regulating and improving students’ emotional, behavioral, or social functioning (26). To our knowledge, five systematic reviews or meta-analyses have examined the effectiveness of resilience programs on the mental health of children or adolescents (13, 14, 27–29). In terms of improving resilience, four studies reported effect sizes ranging from 0.23 to 0.58, while one study did not address this outcome (13). However, these studies have certain limitations. For example, three studies had incomplete search strategies regarding interventions or outcomes (14, 28, 29), and two studies included mixed populations (27, 28). Notably, only one study focused on interventions implemented in school environments (29), which means that the effectiveness of SBIs in improving resilience in children and adolescents still lacks high-quality evidence. The school system possesses sufficient foundational conditions to identify mental health issues effectively in children and adolescents and provide timely onsite interventions (30, 31). Specifically, this system can effectively identify early signs of mental health issues in children and adolescents by implementing universally applicable policies, ensuring access to high-quality educators, and establishing school-based clinics (32). Effective identification is made possible through strong collaboration among healthcare professionals, educators, and school administrators, enabling prompt interventions when these early warning signs emerge. In this way, SBIs not only help alleviate emotional and behavioral disorders in children and adolescents but also ensure that students who have difficulty accessing mental health care receive attention and support (26). Therefore, SBIs play an important role and significance in fostering resilience in children and adolescents.

The protective possibilities framework of resilience suggests that SBIs can promote students’ resilience by providing opportunities for development, as well as emotional, motivational, and strategic support (33). Key elements within the school environment—including teacher behaviors and support in the classroom, peer relationships, and family support and expectations—collectively form a supportive ecosystem that helps students better cope with challenges and stress, thereby enhancing their resilience. Over the past two decades, a series of studies have confirmed the significant efficacy of SBIs in promoting resilience in children and adolescents. For example, a program to support students exposed to trauma was proposed by Amin et al. (34), which includes 10 structured sessions designed to promote resilience by reducing students’ posttraumatic stress disorder symptoms (34). On the basis of the P-A-G-E framework, Cheng et al. (35) proposed the Digital Netizen Alliance program to increase students’ positive coping ability and resilience through the development of positive mental skills (35). Khalsa et al. (36) proposed a yoga program implemented within the school environment, which effectively improved students’ resilience by promoting mindfulness and developing cognitive skills related to self-awareness (36). However, evidence for SBIs in this field is not consistent, with some studies indicating that these interventions have not demonstrated significant efficacy in promoting resilience in children and adolescents (37, 38). In addition, previous studies have indicated that the efficacy of SBIs for children and adolescents may vary depending on population characteristics and intervention implementation. Differences in cognitive abilities, emotional needs, and brain development at various stages of childhood and adolescence may lead to different dose-response relationships in intervention implementation (39). Similarly, factors such as age, cultural background, and socioeconomic status may also contribute to variations in resilience levels (40, 41). Therefore, it is essential to investigate the differential efficacy of interventions based on variations in population characteristics and intervention implementation through moderator analyses. This approach enables the customization of SBIs to accommodate these variations, ensuring more tailored and effective adjustments. In summary, although SBIs can promote the development of resilience in children and adolescents to some extent, the overall efficacy of these interventions in this regard still lacks consistency. For children and adolescents exposed to many stressors, fostering resilience helps them cope positively with stressful situations and enhances their overall well-being (42). Given this, it is necessary to review previous evidence to examine the overall efficacy of SBIs on resilience in children and adolescents. The methodologies used in published related randomized controlled trials (RCTs) vary, resulting in differences in the effect sizes of SBIs on resilience in children and adolescents. This systematic review and meta-analysis aimed to evaluate the overall efficacy of SBIs in promoting resilience in children and adolescents and to provide evidence for advancing mental health care for children and adolescents.

## 2 Methods

This systematic review and meta-analysis followed the Preferred Reporting Items for Systematic Reviews and Meta-Analyses (PRISMA) 2020 guidelines (43) and has been registered in the International Prospective Register of Systematic Reviews (PROSPERO) with the registration number: CRD420251009149.

### 2.1 Search methods

A search was conducted in six electronic databases via Medical Subject Headings and free-text terms: PubMed, Embase, EBSCOhost, Scopus, Web of Science, and APA PsycINFO. In addition, relevant references were manually searched on Google Scholar. The search period ranged from the creation of each database to January 2025. Notably, the included studies must be published in English and have been peer-reviewed. Grey literature is excluded from the literature search, as it may lack rigorous peer review and standardized reporting. The search methods followed the PICOS principles: (P) Population—children or adolescents (age range: 6—19 years); (I) Intervention—school-based interventions (e.g., classroom-based social and emotional learning program, school-based resilience intervention program, school-based emotion regulation program, school-based mindfulness training); (C) Comparator—control groups receiving routine education, wait-list, no-intervention, or active control; (O) Outcome—any assessment for resilience, including trials where it was a primary or secondary outcome; (S) Study design—all types of randomized controlled trials. The search methods of PubMed are presented in **Table 1**. The complete search methods for all electronic databases are detailed in Supplementary material **Table 1**.

**Table 1:** PubMed search strategy.

#1	Resilience[MeSH Terms] OR Resilience[Title/Abstract] OR Resilien*[Title/Abstract]
#2	Intervention*[Title/Abstract] OR Program*[Title/Abstract] OR School intervention*[Title/Abstract] OR School-based*[Title/Abstract] OR School*[Title/Abstract] OR College*[Title/Abstract] OR Universit*[Title/Abstract] OR Campus*[Title/Abstract] OR Classroom*[Title/Abstract] Curricul*[Title/Abstract] OR Educat*[Title/Abstract]
#3	Adolescen*[Title/Abstract] OR Teen*[Title/Abstract] Youth*[Title/Abstract] OR Juven*[Title/Abstract] OR Child*[Title/Abstract] OR Minor*[Title/Abstract] OR Kid[Title/Abstract] OR Kids[Title/Abstract] OR Pediatric*[Title/Abstract] OR Paediatric*[Title/Abstract] OR Pupil*[Title/Abstract] OR Toddler*[Title/Abstract] OR School-age*[Title/Abstract] OR Schoolage*[Title/Abstract]
#4	Randomized controlled trial[Publication Type] OR Randomized[Title/Abstract] OR Placebo[Title/Abstract]
#5	#1 AND #2 AND #3 AND #4

### 2.2 Inclusion and exclusion criteria

The inclusion and exclusion criteria are presented in **Table 2**.

**Table 2:** Inclusion and exclusion criteria.

Category	Inclusion criteria	Exclusion criteria
Population	Children or adolescents (age range: 6—19 years)	Not children or adolescents
Intervention	School-based interventions (e.g., classroom-based social and emotional learning program, school-based resilience intervention program, school-based emotion regulation program, school-based mindfulness training)	Not interventions based on school
Comparator	Control group receiving routine education, wait-list, no-intervention, or active control	No exclusion criteria
Outcome	Any assessment for resilience, including trials where it was a primary or secondary outcome	No exclusion criteria
Study design	All types of randomized controlled trials (RCTs)	Non-randomized controlled trials, such as quasi experiments, observational studies, case reports, study protocols, conference proceedings, review, etc

### 2.3 Study selection and data extraction

The study selection and data extraction were performed by two independent researchers (CYC and ZYM). Following the literature search, all records that fulfilled the predetermined inclusion and exclusion criteria were imported into EndNote 20.6, with duplicates removed. The titles, abstracts, and full texts of the remaining records were independently screened by two researchers. The extracted data included (a) basic information, including the first author, country, and year of publication; (b) participant characteristics, including the type and risk profile of population, mean age (standard deviation), sample size, and percentage of males; (c) details of the interventions and controls; and (d) outcome and measure. Any disagreements arising during the study selection and data extraction processes were addressed through consultation with the corresponding author (SL).

### 2.4 Quality appraisal

The quality appraisal was performed by two independent researchers (CYC and ZYM) via the Revised Cochrane risk-of-bias tool for randomized trials (RoB 2) (44). The RoB 2 examined included studies for potential sources of bias, including the randomization process, deviations from intended interventions, mising outcome data, measurement of the outcome, and selection of the reported result. The quality of the included studies was categorized into three levels: low risk, some concerns, and high risk. Any disagreements arising during the quality appraisal process were addressed through consultation with the corresponding author (SL).

### 2.5 Data synthesis

Given the variations in scales employed in different RCTs and given that the outcome in this study was a continuous variable, the standardized mean difference (SMD; Cohen’s d) combined with 95% confidence intervals (CIs) was used to pool the effect sizes (45). The differences in efficacy between the experimental and control groups were compared via forest plots, and heterogeneity of the pooled results was evaluated via the chi-square test, which is based on the *Q* test and *I^2^* statistic (46). When significant heterogeneity was observed (*I^2^* > 50%, *p* < 0.10), a random-effects model with the DerSimonian-Laird method was employed to pool the effect sizes. In the absence of significant heterogeneity (*I^2^* < 50%, *p* > 0.10), a fixed-effects model with the inverse variance method was employed to pool the effect sizes (45). The sources of heterogeneity in the pooled results were explored through meta-regression (for continuous variables) and subgroup analysis (for categorical variables) (47, 48). After studies at high risk were excluded, a sensitivity analysis was performed via a stepwise elimination method to evaluate the robustness of the pooled results (45). Publication bias was assessed based on a visual inspection of the funnel plot and Egger’s test (49, 50). The trim-and-fill method was used to further evaluate the robustness of the pooled results in the presence of significant publication bias (51). The Grading of Recommendations, Assessment, Development, and Evaluations (GRADE) guidelines were used for the certainty of evidence in the following areas: risk of bias, inconsistency, indirectness, imprecision, and publication bias (52). All data syntheses were conducted using Stata 18.0.

## 3 Results

### 3.1 Search outcomes

**Figure 1:**
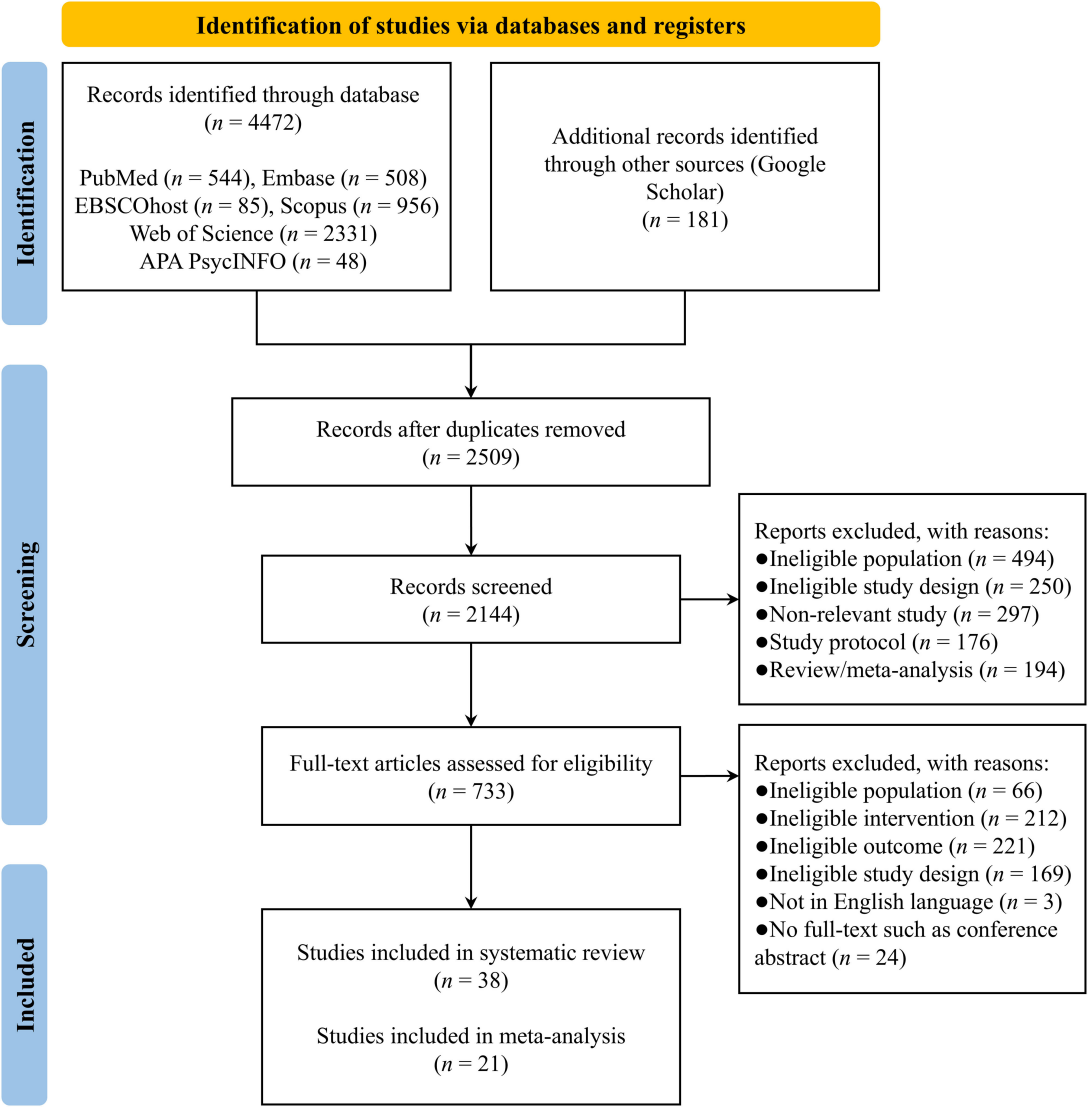
PRISMA flow diagram.

Seven electronic databases were searched, yielding a total of 4,653 records. A systematic identification and manual screening process removed 2,510 duplicates, leaving 2,143 records. A total of 1,411 records were excluded on the basis of titles and abstracts, and 695 records were excluded on the basis of full-text review. Thirty-eight studies were included in the systematic review, with twenty-one studies included in the meta-analysis. Seventeen studies were excluded from the meta-analysis due to a lack of available data (34–38, 53–85) (see **Figure 1**).

### 3.2 Study characteristics

The included studies were published between 2008 and 2024. These studies were conducted in the United States (*n* = 10), China (*n* = 9), Australia (*n* = 4), Pakistan (*n* = 2), India (*n* = 2), and one study each in the United Kingdom, Colombia, Belgium, Italy, Iran, South Korea, Sweden, the Netherlands, Ireland, Spain, and Finland. In terms of population characteristics, 26 studies focused on adolescents, 8 studies focused on children, and the remaining 4 studies included mixed populations. In terms of the risk profile of population, participants in 14 studies came from high-risk groups, which may face additional challenges and difficulties in various aspects, such as lower socioeconomic status, physical and mental health issues, or identities as minority and marginalized groups. The remaining 24 studies involved participants who did not belong to high-risk groups. The experimental group consisted of 8,869 participants, ranging in age from 9.50 to 17.10 years. The control group included a total of 6,861 participants, ranging in age from 9.70 to 17.30 years. In terms of the delivery method of interventions, 36 studies used a group intervention model, while 2 studies employed an individual intervention model. Furthermore, 18 studies employed treatment-as-usual controls, 11 utilized wait-list controls, 6 incorporated no-intervention controls, and 3 applied positive controls (see **Table 3**).

**Table 3:** Main characteristics of included randomized controlled trials.

Study ID	Country	Population		Age (Mean (SD))	Total/M%	Intervention		Comparator	Measurement
		Type	High-risk			Strategy	Delivery method		
Amin et al. (34)	Pakistan	Children	Yes	T: 11.29 (1.48) C: 11.57 (1.41)	T: 38/73.7% C: 37/56.8%	Support for students exposed to Trauma program	Group	No-intervention	CYRM-28
Bogaert et al. (37)	Belgium	Adolescents	No	T: 13.20 (1.00) C: 13.30 (1.08)	T: 95/25.3% C: 93/22.6%	Positive events training	Group	Positive control	CD-RISC-10
Chen et al. (53)	Australia	Children	No	T+C: 10.08 (1.21)	T: 129/NR C: 57/NR	Emotion regulation	Group	Treatment as usual	CYRM-12
Cheng et al. (35)	China	Children	No	T: 10.86 (1.20) C: 10.79 (1.17)	T: 137/NR C: 127/NR	Multicomponent positive psychology program	Individual	Wait-list	CYRM-12
Chisholm et al. (54)	UK	Adolescents	No	T+C: 12.21 (0.58)	T: 354/48.3% C: 303/47.5%	Education plus contact	Group	Treatment as usual	RS-15
Felver et al. (55)	USA	Adolescents	Yes	T: 16.15 (0.90) C: 16.74 (1.17)	T: 16/31.0% C: 11/34.0%	School-based mindfulness intervention	Group	Treatment as usual	SEARS-SF
Gance-Cleveland and Mays (56)	USA	Adolescents	Yes	T: 15.45 (1.24) C: 15.16 (1.14)	T: 49/26.5% C: 42/52.4%	School-based Support Groups	Group	Wait-list	HDLF-Y
Gao et al. (57)	China	Adolescents	No	T: NR C: NR	T: 84/53.6% C: 83/45.8%	Positive education intervention based on the PERMA	Group	Treatment as usual	CD-RISC-25
Gomez-Restrepo et al. (58)	Colombia	Adolescents	Yes	T: 14.62 (2.04) C: 14.85 (2.39)	T: 50/36.0% C: 20/65.0%	Digital app-supported and evidence-based intervention	Group	Treatment as usual	CD-RISC-25
Greco et al. (59)	Italy	Adolescents	No	T: 14.50 (0.70) C: 14.60 (0.70)	T: 48/50.0% C: 50/50.0%	Karate-based intervention program	Group	Wait-list	CYRM-28
Green et al. (60)	USA	Adolescents	No	T: 12.30 (NR) C: 12.40 (NR)	T: 188/67.2% C: 177/64.2%	Classroom-based social and emotional learning program	Group	Treatment as usual	RSCA
Green et al. (61)	USA	Children	Yes	T: 9.50 (NR) C: 9.70 (NR)	T: 47/55.3% C: 47/51.1%	Classroom-based social and emotional learning program	Group	Treatment as usual	RSCA
Green et al. (62)	USA	Adolescents	No	T+C: 15.7 (NR)	T+C: 372/48.0%	Classroom-based social and emotional learning program	Group	Treatment as usual	RSCA
Hatamizadeh et al. (63)	Iran	Adolescents	Yes	T: NR C: NR	T: 61/52.5% C: 61/68.9%	School-based resilience intervention program	Group	Treatment as usual	CD-RISC-25
Ho et al. (64)	China	Adolescents	No	T: 12.32 (0.76) C: 12.26 (0.75)	T: 333/42.6% C: 331/41.1%	Positive youth development-based sports mentorship program	Group	Positive control	CD-RISC-25
Huang et al. (65)	China	Children	No	T: 9.90 (0.54) C: 9.96 (0.54)	T: 391/56.3% C: 384/52.1%	Resilience-focused intervention	Group	Wait-list	RSCA
Hyun et al. (66)	Korea	Adolescents	Yes	T: 12.60 (0.51) C: 12.47 (0.52)	T: 17/100.0% C: 17/100.0%	School-based cognitive-behavioral therapy	Group	Wait-list	KARS
Irfan Arif and Mirza (67)	Pakistan	Adolescents	Yes	T: NR C: NR	T: 32/100.0% C: 32/100.0%	Programme based on a resilience building module for teachers	Group	Treatment as usual	RAS
Johnstone et al. (68)	Australia	Children	No	T+C: 11.04 (1.40)	T: 185/NR C: 25/NR	School-based emotion regulation program	Group	Treatment as usual	CYRM‐12
Jones and Destin (69)	USA	Adolescents	No	T: NR C: NR	T + C: 350/57.0%	Expressive writing exercises	Group	Treatment as usual	Self-reported measure
Khalsa et al. (36)	USA	Adolescents	No	T: 16.80 (0.60) C: 16.90 (0.80)	T: 74/54.1% C: 47/63.8%	Yoga sessions based upon the Yoga Ed program	Group	Treatment as usual	RS-25
Laundy et al. (70)	Sweden	Children	Yes	T: 11.70 (1.63) C: 11.20 (1.96)	T: 22/36.4% C: 12/25.0%	Training for mindfulness and resilience	Group	Positive control	RS-10
Leventhal et al. (71)	India	Adolescents	Yes	T: 13.01 (1.16) C: 12.94 (1.18)	T: 1730/0.0% C: 737/0.0%	Resilience-based program	Group	No-intervention	CD-RISC-10
Li et al. (72)	China	Adolescents	No	T: 14.07 (0.51) C: 13.80 (0.56)	T: 38/39.5% C: 38/39.5%	Group counseling using the Achieving Success Everyday model	Group	No-intervention	RSCA-27
Liu et al. (73)	China	Adolescents	No	T: 15.86 (0.55) C: 15.92 (0.38)	T: 57/40.4% C: 60/43.3%	School-based mindfulness training	Group	No-intervention	RSCA-27
Llistosella et al. (74)	Spain	Adolescents	Yes	T: NR C: NR	T: 255/52.5% C: 323/53.6	School-based resilience intervention	Group	Wait-list	CYRM-32
Mertens et al. (75)	Netherlands	Adolescents	No	T: 12.35 (0.61) C: 12.47 (0.64)	T: 925/49.9% C: 374/53.2%	Rock and Water lessons	Group	Treatment as usual	CD-RISC-3
Moore et al. (76)	Australia	Adolescents	No	T+C: 12.76 (0.68)	T+C: 283/48.0%	Martial arts-based psychosocial interventions	Group	Wait-list	CYRM-28
Moran et al. (77)	China	Adolescents	No	T: 11.60 (0.52) C: 11.60 (0.53)	T: 160/58.8% C: 70/48.6%	School-based health coaching intervention with a mindfulness component	Individual	No-intervention	CYRM-17
Niu et al. (38)	China	Adolescents	Yes	T: NR C: NR	T: 28/NR C: 28/NR	Culturally-attuned resilience intervention	Group	Treatment as usual	CD-RISC-25
Noggle et al. (78)	USA	Adolescents	No	T: 17.10 (0.60) C: 17.30 (0.80)	T: 36/39.0% C: 15/53.0%	Kripalu-based yoga program	Group	Treatment as usual	RS-25
O'Connor et al. (79)	Ireland	Adolescents	No	T: 11.04 (0.68) C: 11.09 (0.67)	T: 262/40.5% C: 342/40.1%	Process-based cognitive-behavioral therapy	Group	Wait-list	CD-RISC-10
Peter et al. (80)	India	Adolescents	Yes	T: 13.05 (0.67) C: 13.10 (0.57)	T: 33/45.5% C: 32/50.0%	Mindfulness-based cognitive therapy	Group	Wait-list	BURS
Rice et al. (81)	Australia	Children and adolescents	No	T: NR C: NR	T: 89/NR C: 28/NR	Emotion regulation program	Group	Treatment as usual	CYRM-12
Rich et al. (82)	USA	Children	Yes	T: 10.83 (0.59) C: 10.82 (0.53)	T: 82/65.9% C: 87/63.2%	Resilience builder program	Group	Wait-list	RSCA-64
Seale et al. (83)	USA	Children and adolescents	No	T: 11.30 (0.90) C: 11.50 (1.00)	T: 343/43.4% C: 200/46.3%	Character-based resilience curriculum	Group	Wait-list	CD-RISC-10
Tang et al. (84)	China	Children and adolescents	No	T: 12.27 (1.60) C: 12.67 (1.71)	T: 732/51.6% C: 881/49.7%	Peer education	Group	No-intervention	RSCA-27
Volanen et al. (85)	Finland	Children and adolescents	No	T: NR C: NR	T: 1232/NR C: 1202/NR	Mindfulness-based interventions	Group	Treatment as usual	RS-14

T, Test group; C, Control group; M%, Percentage of boys; CYRM, Child and Youth Resilience Measure; CD-RISC, Connor-Davidson Resilience Scale; RS, Resilience Scale; SEARS-SF, Social-Emotional Assets and Resilience Scales; HDLF-Y, Health and Daily Living Form for Youth; RSCA, Resiliency Scales for Children and Adolescents; KARS, Korean Adolescent Resilience Scale; RAS, Resilience Assessment Scale; BURS, Bharathiar University Resilience Scale.

### 3.3 Quality appraisal

**Figure 2:**
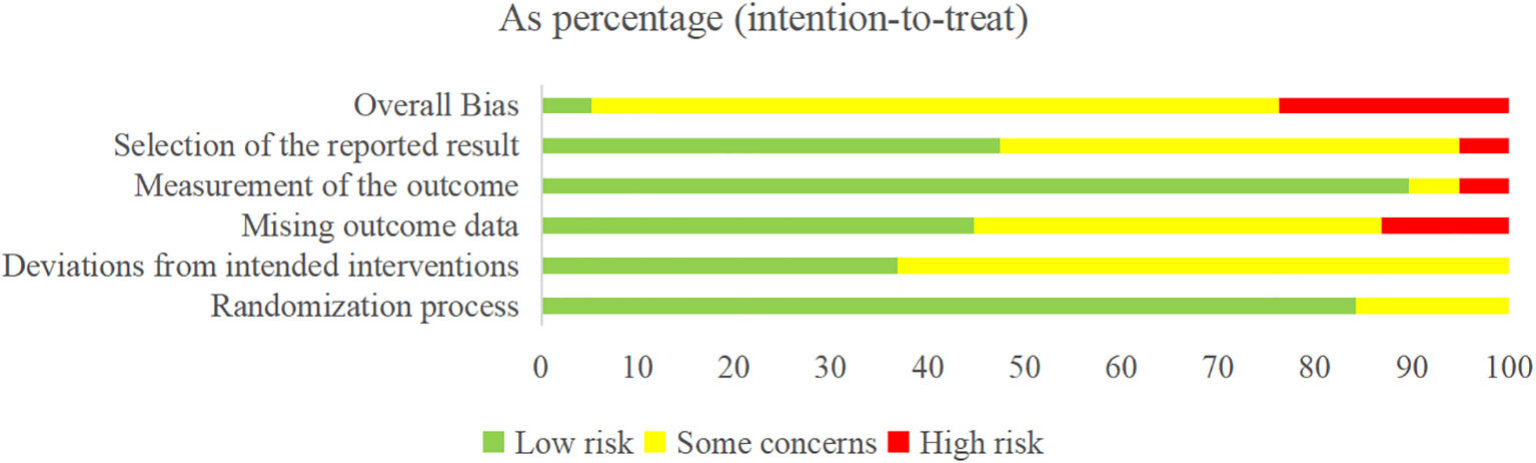
Risk of bias summary.

The risk of bias ranged from low to high (see **Figure 2**, **Table 4**). For the randomization process, 6 studies were assessed as having some concerns due to baseline differences, while the remaining studies as being of low risk. For the deviations from intended interventions, 24 studies were assessed as having some concerns due to the use of inappropriate analysis to estimate the effect of assignment to intervention, while the remaining studies as being of low risk. For the mising outcome data, 21 studies were assessed as having some concerns or high risk due to missing data for some participants and a lack of evidence that the result was not biased by missing outcome data, while the remaining studies as being of low risk. For the measurement of the outcome, 4 studies were assessed as having some concerns or high risk due to the method of measuring the outcome inappropriate, while the remaining studies as being of low risk. For the selection of the reported result, 20 studies were assessed as having some concerns or high risk due to multiple eligible outcome measurements within the outcome domain, while the remaining studies as being of low risk.

**Table 4:** Risk of bias summary for the included effect estimates.

Study ID	D1	D2	D3	D4	D5	Overall bias
Amin et al. (34)	Some concerns	Some concerns	Low	Low	Low	Some concerns
Bogaert et al. (37)	Low	Low	Low	Low	Some concerns	Some concerns
Chen et al. (53)	Low	Some concerns	Some concerns	Low	High	High
Cheng et al. (35)	Low	Some concerns	Some concerns	Low	Low	Some concerns
Chisholm et al. (54)	Low	Low	Some concerns	Low	Low	Some concerns
Felver et al. (55)	Low	Low	High	Some concerns	Low	High
Gance-Cleveland and Mays (56)	Some concerns	Low	Low	High	Low	High
Gao et al. (57)	Low	Some concerns	Low	Low	Some concerns	Some concerns
Gomez-Restrepo et al. (58)	Low	Some concerns	Some concerns	Low	Low	Some concerns
Greco et al. (59)	Low	Some concerns	Low	Low	Low	Some concerns
Green et al. (60)	Low	Some concerns	Low	Low	Low	Some concerns
Green et al. (61)	Some concerns	Some concerns	Some concerns	Low	Low	Some concerns
Green et al. (62)	Some concerns	Some concerns	Some concerns	Low	Low	Some concerns
Hatamizadeh et al. (63)	Low	Some concerns	Low	Low	Some concerns	Some concerns
Ho et al. (64)	Low	Low	Low	Low	Low	Low
Huang et al. (65)	Low	Low	Low	Low	Some concerns	Some concerns
Hyun et al. (66)	Low	Some concerns	High	Low	Low	High
Irfan Arif and Mirza (67)	Low	Some concerns	Low	Low	Some concerns	Some concerns
Johnstone et al. (68)	Low	Some concerns	High	Low	Some concerns	High
Jones and Destin (69)	Low	Some concerns	Low	Some concerns	Some concerns	Some concerns
Khalsa et al. (36)	Low	Some concerns	Low	Low	Some concerns	Some concerns
Laundy et al. (70)	Low	Some concerns	High	Low	Some concerns	High
Leventhal et al. (71)	Low	Some concerns	Some concerns	Low	Some concerns	Some concerns
Li et al. (72)	Low	Some concerns	Low	Low	Some concerns	Some concerns
Liu et al. (73)	Low	Some concerns	Some concerns	Low	Low	Some concerns
Llistosella et al. (74)	Low	Low	Some concerns	Low	Some concerns	Some concerns
Mertens et al. (75)	Low	Low	Some concerns	Low	Some concerns	Some concerns
Moore et al. (76)	Low	Low	Some concerns	Low	Low	Some concerns
Moran et al. (77)	Low	Low	Some concerns	Low	Low	Some concerns
Niu et al. (38)	Low	Some concerns	Low	Low	Low	Some concerns
Noggle et al. (78)	Low	Low	Low	Low	Low	Low
O'Connor et al. (79)	Low	Low	Some concerns	Low	Some concerns	Some concerns
Peter et al. (80)	Some concerns	Some concerns	Low	High	Some concerns	High
Rice et al. (81)	Low	Some concerns	High	Low	Some concerns	High
Rich et al. (82)	Some concerns	Some concerns	Some concerns	Low	Some concerns	Some concerns
Seale et al. (83)	Low	Low	Some concerns	Low	High	High
Tang et al. (84)	Low	Some concerns	Low	Low	Low	Some concerns
Volanen et al. (85)	Low	Low	Some concerns	Low	Some concerns	Some concerns

D1, Randomization process; D2, Deviations from intended interventions; D3, Mising outcome data; D4, Measurement of the outcome; D5, Selection of the reported result.

### 3.4 Pairwise meta-analyses

**Figure 3:**
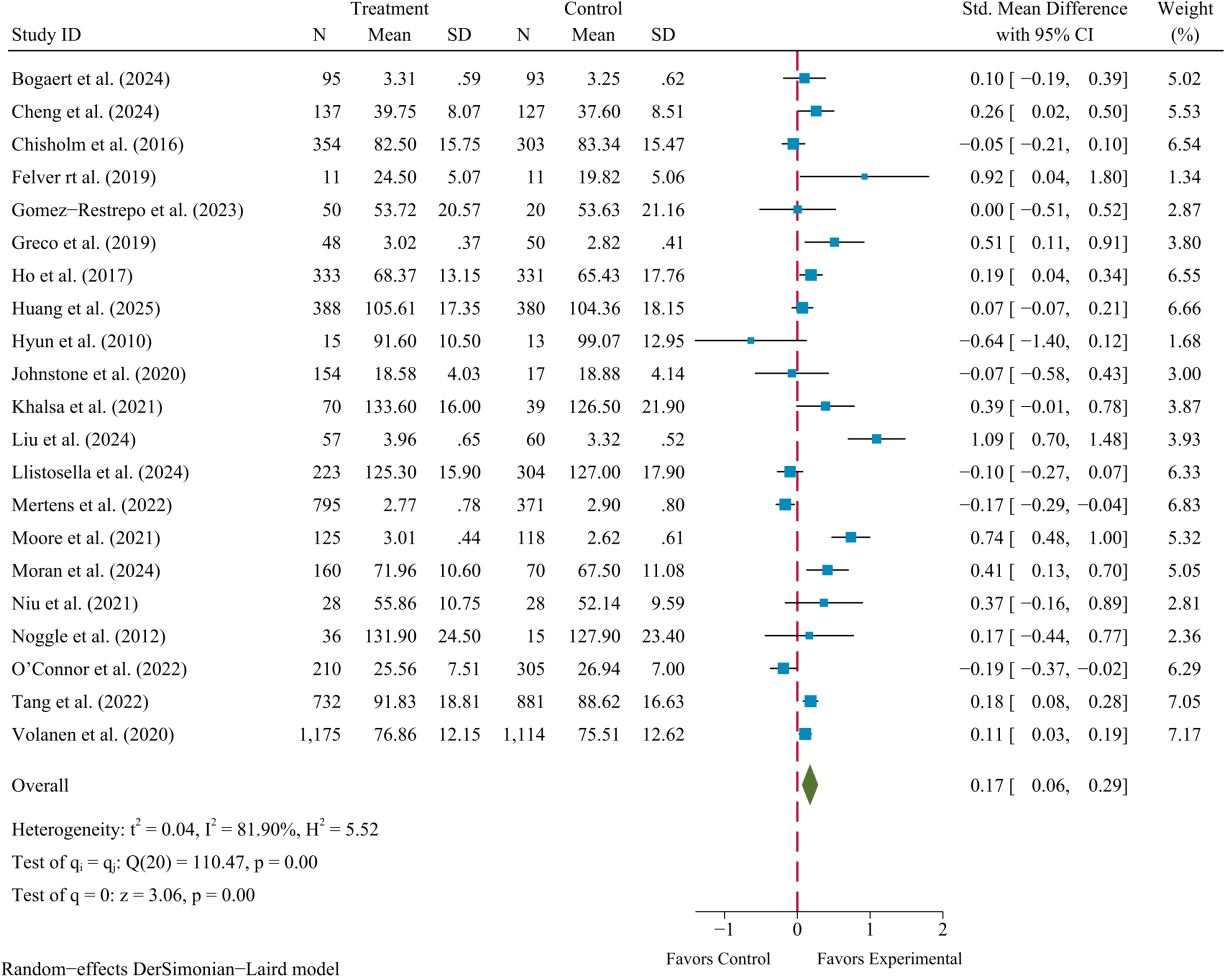
Forest plot of the effects of school-based interventions on resilience.

The pairwise meta-analyses included 21 RCTs with available data. Evidence for a small effect of SBIs on resilience in children and adolescents observed (SMD = 0.17, 95% Cl 0.06–0.29, *p* < 0.01), with considerable heterogeneity identified (*I^2^* = 81.90%, *Q* = 110.47, *p* < 0.01). The corresponding forest plot for the pooled results is presented in **Figure 3**.

### 3.5 Moderator analyses

Based on meta-regression to test the moderating effects, the results indicated that year of publication (*p* = 0.25), total sample size (*p* = 0.17), length of intervention (*p* = 0.76), frequency of intervention (*p* = 0.38), and duration of intervention (*p* = 0.59) did not moderate the overall efficacy of SBIs on resilience in children and adolescents. Based on subgroup analysis to test the moderating effects, the results indicated that the type of intervention groups exhibited significant differences in the pooled results (*Q_b_* (4) *=* 15.60, *p* < 0.01). In contrast, no significant differences were observed in the pooled results with respect to the type of control groups (*Q_b_* (3) = 3.64, *p =* 0.30), type of population (*Q_b_* (1) = 0.66, *p* = 0.42), risk profile of population (*Q_b_* (1) *=* 0.56, *p =* 0.45), and risk of bias (*Q_b_* (2) = 0.16, *p =* 0.92) (see **Table 5**).

**Table 5:** Moderator analyses for resilience.

Continuous variables	Studies	*β*	SE	t	*p*
Sociodemographic characteristics					
Year of publication	20	0.03	0.02	1.19	0.25
Total sample sizes	20	-0.01	0.01	-1.46	0.17
Implementation parameters					
Length of intervention	20	-0.01	0.02	-0.32	0.76
Frequency of intervention	20	-0.06	0.07	-0.91	0.38
Duration of intervention	20	0.01	0.01	0.55	0.59

Categorical variables	Studies	SMD [95% CI]			*p*
Type of intervention groups					
Sport-focused SBIs	5	0.41 [0.15, 0.67]			< 0.01
Resilience-focused SBIs	4	0.09 [-0.08, 0.26]			0.32
Mindfulness-focused SBIs	4	0.57 [0.10, 1.04]			0.02
CBT-focused SBIs	2	-0.26 [-0.56, 0.05]			0.10
Other-component-focused SBIs	6	0.01 [-0.16, 0.17]			0.96
Test of group differences	*Q_b_* (4) *=* 15.60		*p* < 0.01		
Type of control groups					
No-intervention	3	0.53 [0.07, 0.99]			0.03
Wait-list	8	0.12 [-0.11, 0.36]			0.30
Treatment as usual	8	0.07 [-0.09, 0.23]			0.38
Active control (excluding TAU)	2	0.17 [0.03, 0.30]			0.01
Test of group differences	*Q_b_* (3) *=* 3.64		*p =* 0.30		
Type of population					
Children (6—12 years)	3	0.11 [-0.02, 0.25]			0.11
Adolescents (12—19 years)	18	0.19 [0.06, 0.32]			< 0.01
Test of group differences	*Q_b_* (1) *=* 0.66		*p* = 0.42		
Risk profile of population					
High-risk	5	0.06 [-0.29, 0.41]			0.75
Non high-risk	16	0.20 [0.08, 0.32]			< 0.01
Test of group differences	*Q_b_* (1) *=* 0.56		*p* = 0.45		
Risk of bias					
Low risk	3	0.19 [0.04, 0.33]			0.01
Some concerns	3	0.19 [0.06, 0.31]			< 0.01
High risk	15	0.03 [-0.73, 0.79]			0.93
Test of group differences	*Q_b_* (2) *=* 0.16		*p* = 0.92		

TAU, Treatment as usual; *β*, Regression coefficient; SE, Standard error; SMD, Standardized mean difference; CI, Confidence interval; *Q*, Cochran’s *Q* statistic with *p* value; SBIs, School-based interventions; CBT, Cognitive-behavioral therapy.

### 3.6 Sensitivity analyses

After studies at high risk were excluded, sensitivity analyses were performed via a stepwise elimination method. The results indicated that of the pooled results remained consistent despite variations in study selection, suggesting that the overall efficacy of SBIs on resilience in children and adolescents was robust, with an effect size ranging from 0.14 to 0.21 (see **Table 6**).

**Table 6:** Sensitivity analyses for outcomes by omitting individual studies.

Outcome	Study omitted	SMD	95% CI	
			Lower bound	Upper bound
Resilience	Bogaert et al. (37)	0.19	0.07	0.31
	Cheng et al. (35)	0.18	0.06	0.30
	Chisholm et al. (54)	0.21	0.08	0.33
	Gomez-Restrepo et al. (58)	0.19	0.08	0.31
	Greco et al. (59)	0.17	0.06	0.29
	Ho et al. (64)	0.19	0.07	0.31
	Huang et al. (65)	0.20	0.07	0.32
	Khalsa et al. (36)	0.18	0.06	0.29
	Liu et al. (73)	0.14	0.04	0.24
	Llistosella et al. (74)	0.21	0.09	0.33
	Mertens et al. (75)	0.21	0.10	0.32
	Moore et al. (76)	0.14	0.04	0.25
	Moran et al. (77)	0.17	0.06	0.29
	Niu et al. (38)	0.18	0.06	0.30
	Noggle et al. (78)	0.19	0.07	0.30
	O'Connor et al. (79)	0.21	0.10	0.33
	Tang et al. (84)	0.19	0.06	0.32
	Volanen et al. (85)	0.20	0.07	0.33
	Combined	0.18	0.06	0.29

SMD, Standardized mean difference; CI, Confidence interval.

### 3.7 Publication bias and certainty of evidence

**Figure 4:**
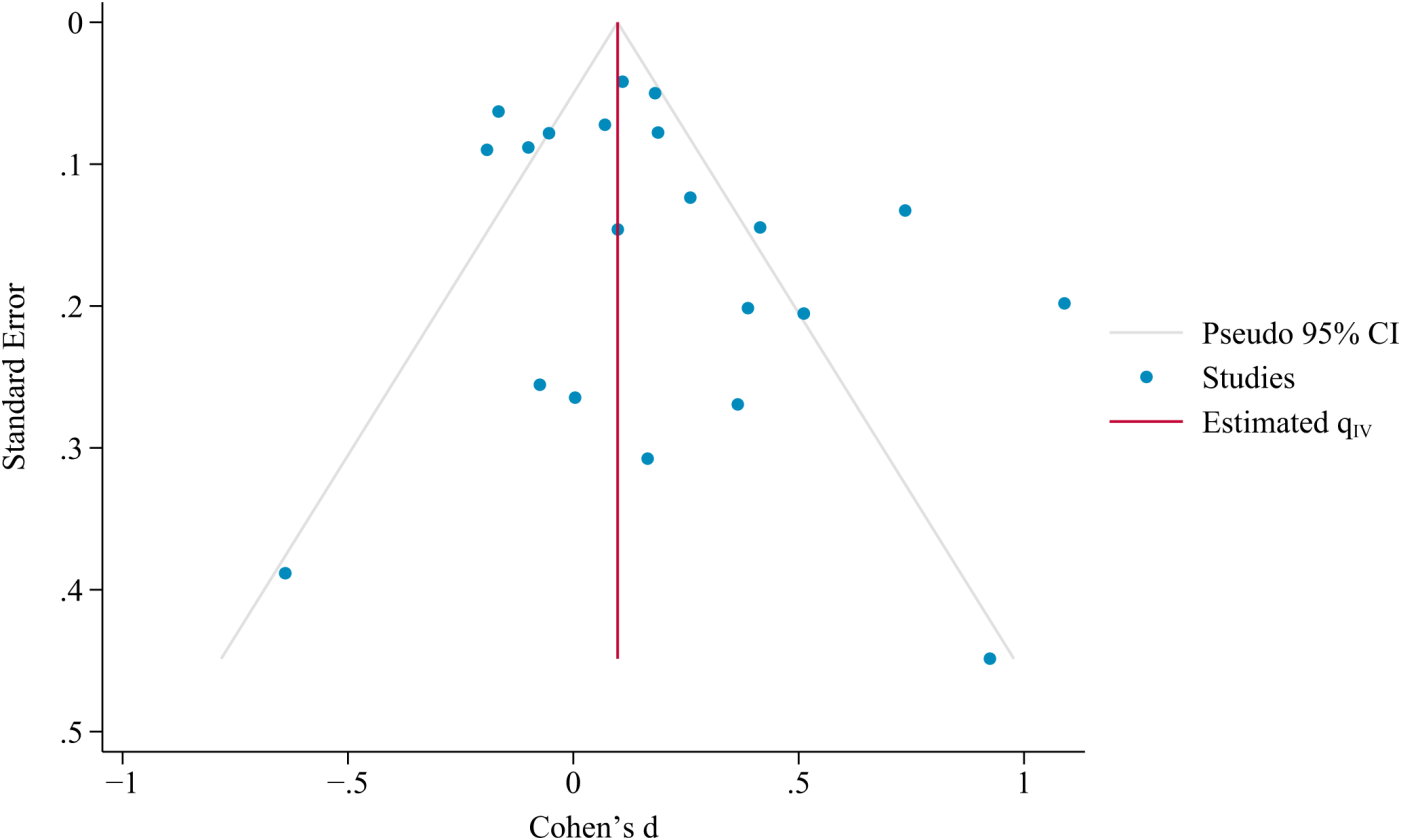
Funnel plot of publication bias for resilience.

Publication bias was assessed based on a visual inspection of the funnel plot and Egger’s test. The results indicated that the included studies were evenly distributed on both sides of the funnel plot (see **Figure 4**), with an Egger’s test *p* of 0.1292. This finding indicates that publication bias has no influence on this study, and there is no significant systematic association between effect sizes and standard errors. According to the GRADE ratings, the certainty of evidence for resilience was very low (see **Table 7**).

**Table 7:** Certainty of evidence rating (GRADE).

GRADE criteria	Rating	Certainty of evidence
Risk of bias	No downgrade. Only 23.7% high risk of bias.	⊕⊝⊝⊝ Very low
Inconsistency	Downgrade by two levels due to considerable inconsistency identified (*I^2^* = 81.90%).	
Indirectness	Downgrade by one level due to indirectness of participants and interventions.	
Imprecision	No concerns (SMD = 0.17, 95% Cl 0.06-0.29).	
Publication bias	No publication bias is suspected.	

SMD, Standardized mean difference; CI, Confidence interval; *I^2^*, Heterogeneity index in percentage.

## 4 Discussion

Children and adolescents often face various stressors as they grow up and may experience cognitive, emotional, and behavioral disorders, particularly when encountering trauma or adversity. Fostering resilience in children and adolescents helps them adapt positively and effectively cope with stressful situations, reducing the risk of maladaptive psychological and behavioral responses and thereby promoting the development of their physical and mental health (86). This systematic review and meta-analysis aimed to evaluate the overall efficacy of SBIs in promoting resilience in children and adolescents and to provide evidence for advancing mental health care for children and adolescents. The pairwise meta-analyses indicated that SBIs significantly enhanced resilience in children and adolescents compared to the control group (SMD = 0.17, 95% Cl 0.06–0.29, *p* < 0.01). The certainty of evidence for resilience was very low on the basis of the GRADE ratings (52). This finding is supported by previous systematic reviews and meta-analyses (29, 87).

Globally, 10—20% of children and adolescents are experiencing mental health issues (88), and only a minority of these children and adolescents have access to medical-level care due to limited medical resources. These findings suggest that non-pharmacological interventions may have important potential in addressing these challenges. Schools are environments that children and adolescents rely on for their development. Implementing interventions within the school environment can effectively reduce various barriers, including family financial burdens, caregiver burdens, transportation needs, and limited insurance coverage, without requiring significant additional time and human resources. More importantly, schools provide a familiar environment for students, along with support from teachers and peers, which is particularly important for promoting children’s and adolescents’ acceptance and adaptation to interventions (89–91). Furthermore, SBIs are especially crucial for children and adolescents from impoverished or minority communities, as they may be academically disadvantaged compared to children from non-poor or white families. Fostering resilience through SBIs provides these groups with higher-quality and more comprehensive educational experience, helping to prevent inequities in interventions caused by certain disparities (92).

In terms of pairwise meta-analyses, the pooled results indicated that SBIs significantly enhanced resilience in children and adolescents. The robustness of this finding was confirmed by sensitivity analyses, but it was accompanied by considerable heterogeneity. Although this study was unable to identify the sources of heterogeneity in the pooled results through meta-regression and subgroup analyses, this heterogeneity may reflect differences in population characteristics and intervention implementation. On the one hand, the differential efficacy of SBIs may be due to different health challenges or school environments experienced by children and adolescents. On the other hand, the specific processes, measures, and dosage of intervention implementation may also contribute to variations in efficacy. More importantly, the high heterogeneity may reveals a critical issue in the design of current interventions—namely, the lack of standardized procedures, theory-driven, and scalability. The absence of these elements not only limits the comparability and replicability of research findings, but also undermines the potential for SBIs to be effectively scaled and implemented in real educational settings. Researchers and practitioners should strive to develop clear intervention manuals and operational guidelines tailored to the specific conditions of each school, covering key elements such as goal setting, activity content, intervention arrangements, and required resources. The protective possibilities framework of resilience may hold promise in addressing this challenge (33). Future research could establish a unified intervention process and indicator system based on this framework, which would help ensure quality control in intervention implementation. In term of the scalability of SBIs, priority should be given to their cost-effectiveness, feasibility, and adaptability. Although the overall effect size of this study is small, its clinical efficacy may be influenced by population characteristics and intervention implementation. It is recommended to involve educational practitioners (e.g., teachers) during the intervention development stage to ensure that the intervention content aligns with students’ realities and provides implementation willingness and local adaptability. For children and adolescents facing multiple stressors, identifying promising and targeted interventions to enhance their resilience is particularly crucial. In light of this, the differential efficacy of SBIs should be further explored in enhancing resilience in children and adolescents, and identify the most effective SBI should be identified on the basis of the specific conditions of children and adolescents. This will help maximize the efficacy of SBIs in enhancing resilience in children and adolescents in the context of limited medical resources.

The findings of this study should be interpreted with consideration of its limitations. First, the outcome of this study focused only on resilience and did not address other potential outcomes similar to resilience, such as mental toughness and grittiness, although SBIs may have a homogenous effect on these outcomes. Second, this study primarily examined the overall efficacy of SBIs on resilience in children and adolescents and did not identify the most promising interventions. It is recommended that future research should build on existing evidence by conducting more high-quality RCTs and network meta-analyses to explore the differential efficacy of various SBIs in enhancing resilience in children and adolescents. Finally, although this study conducted moderator analyses, the results did not reveal potential sources of heterogeneity. Future research could explore the heterogeneity of the pooled results by incorporating more comprehensive information on demographic characteristics and intervention implementation. This helps further expand insights into the role of SBIs in enhancing resilience, especially in contexts with limited medical resources.

## 5 Conclusions

In conclusion, the results of this study suggest that SBIs have a positive effect on the resilience of children and adolescents. In the context of limited medical resources, SBIs could serve as a complementary or alternative therapy to promote the ability of children and adolescents to adapt to stressors. Given the considerable heterogeneity identified, clinical practice should prioritize the selection and implementation of interventions that take into account the demographic characteristics of children and adolescents. These interventions should be personalized on the basis of actual dose-response to improve the overall well-being of children and adolescents and reduce the risk of maladaptive psychological and behavioral responses.
