# Supplementary material for: School-based interventions for resilience in children and adolescents: a systematic review and meta-analysis of randomized controlled trials
Source: Front Psychiatry. 2025 May 19;16:1594658. doi: 10.3389/fpsyt.2025.1594658 (PMC12127306; doi:10.3389/fpsyt.2025.1594658)
Supplement: Supplementary file 1 [file DataSheet1.docx]

Supplementary Material

# Supplementary Figures and Tables

## Supplementary Tables

| **Supplementary Table 1. Search strategy** | | |
| --- | --- | --- |
| Electronic database | Search strategy | Retrieved result |
| PubMed | ((((Resilience[MeSH Terms]) OR (Resilience[Title/Abstract] OR Resilien*[Title/Abstract])) AND (Intervention*[Title/Abstract] OR Program*[Title/Abstract] OR School intervention*[Title/Abstract] OR School-based*[Title/Abstract] OR School*[Title/Abstract] OR College*[Title/Abstract] OR Universit*[Title/Abstract] OR Campus*[Title/Abstract] OR Classroom*[Title/Abstract] OR Curricul*[Title/Abstract] OR Educat*[Title/Abstract])) AND (Adolescen*[Title/Abstract] OR Teen*[Title/Abstract] OR Youth*[Title/Abstract] OR Juven*[Title/Abstract] OR Child*[Title/Abstract] OR Minor*[Title/Abstract] OR Kid[Title/Abstract] OR Kids[Title/Abstract] OR Pediatric*[Title/Abstract] OR Paediatric*[Title/Abstract] OR Pupil*[Title/Abstract] OR Toddler*[Title/Abstract] OR School-age*[Title/Abstract] OR Schoolage*[Title/Abstract])) AND ((Randomized controlled trial[Publication Type]) OR (Randomized[Title/Abstract] OR Placebo[Title/Abstract])) | 544 |
| Embase | (resilience:ti,ab,kw OR resilien*:ti,ab,kw) AND (intervention*:ti,ab,kw OR program*:ti,ab,kw OR 'school intervention*':ti,ab,kw OR 'school based*':ti,ab,kw OR school*:ti,ab,kw OR college*:ti,ab,kw OR universit*:ti,ab,kw OR campus*:ti,ab,kw OR classroom*:ti,ab,kw OR curricul*:ti,ab,kw OR educat*:ti,ab,kw) AND (adolescen*:ti,ab,kw OR teen*:ti,ab,kw OR youth*:ti,ab,kw OR juven*:ti,ab,kw OR child*:ti,ab,kw OR minor*:ti,ab,kw OR kid:ti,ab,kw OR kids:ti,ab,kw OR pediatric*:ti,ab,kw OR paediatric*:ti,ab,kw OR pupil*:ti,ab,kw OR toddler*:ti,ab,kw OR 'school age*':ti,ab,kw OR schoolage*:ti,ab,kw) AND ('randomized controlled trial':ti,ab,kw OR randomized:ti,ab,kw OR placebo:ti,ab,kw) | 508 |
| EBSCOhost | SU (Resilience OR Resilien*) AND SU (Intervention* OR Program* OR School intervention* OR School-based* OR School* OR College* OR Universit* OR Campus* OR Classroom* OR Curricul* OR Educat*) AND SU (Adolescen* OR Teen* OR Youth* OR Juven* OR Child* OR Minor* OR Kid OR Kids OR Pediatric* OR Paediatric* OR Pupil* OR Toddler* OR School-age* OR Schoolage*) AND SU (Randomized controlled trial OR Randomized OR Placebo) | 85 |
| Scopus | ( TITLE-ABS-KEY ( "Resilience" OR "Resilien*" ) AND TITLE-ABS-KEY ( "Intervention*" OR "Program*" OR "School intervention*" OR "School-based*" OR "School*" OR "College*" OR "Universit*" OR "Campus*" OR "Classroom*" OR "Curricul*" OR "Educat*" ) AND TITLE-ABS-KEY ( "Adolescen*" OR "Teen*" OR "Youth*" OR "Juven*" OR "Child*" OR "Minor*" OR "Kid" OR "Kids" OR "Pediatric*" OR "Paediatric*" OR "Pupil*" OR "Toddler*" OR "School-age*" OR "Schoolage*" ) AND TITLE-ABS-KEY ( "Randomized controlled trial" OR "Randomized" OR "Placebo" ) ) | 956 |
| Web of Science | (((TS=(Resilience OR Resilien*)) AND TS=(Intervention* OR Program* OR School intervention* OR School-based* OR School* OR College* OR Universit* OR Campus* OR Classroom* OR Curricul* OR Educat*)) AND TS=(Adolescen* OR Teen* OR Youth* OR Juven* OR Child* OR Minor* OR Kid OR Kids OR Pediatric* OR Paediatric* OR Pupil* OR Toddler* OR School-age* OR Schoolage*)) AND TS=(Randomized controlled trial OR Randomized OR Placebo) and Preprint Citation Index (Exclude – Database) | 2331 |
| APA PsycINFO | Any Field: Resilience OR Any Field: Resilien* AND Any Field: Intervention* OR Any Field: Program* OR Any Field: School intervention* OR Any Field: School-based* OR Any Field: School* OR Any Field: College* OR Any Field: Universit* OR Any Field: Campus* OR Any Field: Classroom* OR Any Field: Curricul* OR Any Field: Educat* AND Any Field: Adolescen* OR Any Field: Teen* OR Any Field: Youth* OR Any Field: Juven* OR Any Field: Child* OR Any Field: Minor* OR Any Field: Kid OR Any Field: Kids OR Any Field: Pediatric* OR Any Field: Paediatric* OR Any Field: Pupil* OR Any Field: Toddler* OR Any Field: School-age* OR Any Field: Schoolage* AND Any Field: Randomized controlled trial OR Any Field: Randomized OR Any Field: Placebo | 48 |
